# Analysis of the source of aggressiveness in gamecocks

**DOI:** 10.1038/s41598-020-63961-1

**Published:** 2020-04-24

**Authors:** Tomoyoshi Komiyama, Masanobu Yoshikawa, Keiko Yokoyama, Hiroyuki Kobayashi

**Affiliations:** 1 Department of Clinical Pharmacology, Tokai University School of Medicine, 143 Shimokasuya, Isehara, Kanagawa 259-1193 Japan; 2 Support Center for Medical Research and Education, Tokai University, 143 Shimokasuya, Isehara, Kanagawa 259-1193 Japan

**Keywords:** Animal breeding, Behavioural genetics, Molecular biology

## Abstract

Although the fighting behaviour in gamecocks has evolved because of artificial selection, it is unknown whether the selection for aggressiveness affects neurotransmitter levels in the avian central nervous system. We sought to identify the source and origin of this trait. We collected the brain samples from 6 female Shamo gamecocks and 5 Shaver Brown chickens (control; bred for egg production). The midbrain levels of norepinephrine (NE) were significantly higher in Shamo gamecocks (*P* = 0.0087) than in the controls. Moreover, alleles encoding adrenergic receptors differed between the breeds in terms of response to NE. Gene mutations specific to Shamo and potentially associated with fighting behaviour were in sites T440N of *ADRα1D*; V296I of *ADRα2A*; and T44I, Q232R, and T277M of *ADRβ2*. The evolutionary analysis indicated that the ADRβ2 (T44I and Q232R) mutations were heritable in all Galliformes, whereas the T440N mutation of ADRα1D and V296I mutations of ADRα2A were unique to Shamo and originated by artificial selection. A high NE level may confer a selective advantage by enabling gamecocks to be aggressive and pain tolerant. Therefore, the strong fighting behaviour of Shamo has resulted from a combination of naturally inherited and mutant genes derived by artificial selection.

## Introduction

Seventeen varieties of domesticated chicken (*Gallus gallus domesticus*) known as Japanese ornamental chickens were developed by artificial selection over a long period for their cultural entertainment value. They are characterised by different body colours and shapes^1–5^. Shamo gamecocks were bred for fighting. The birds were artificially selected under extreme stress to isolate desirable traits such as strength, aggression, and anxiety and pain endurance during cockfighting^3,4^. The shape and colour characteristics of modern-day Japanese Shamo have been depicted in wildlife caricatures approximately 1000 y ago. Shamo have body shapes and fighting styles distinct from those of other chickens^5,6^. After a match, losing Shamo males are euthanised by the breeders, whereas the victorious males are bred with healthy Shamo females. These pedigreed lines have been passed down from generation to generation and are highly valuable to breeders^2^.

The objective of the present study was to elucidate the source of the fighting behaviour in Shamo gamecocks. Our previous molecular evolutionary studies based on the mitochondrial D-loop region revealed that the Shamo gamecock breed originated from red junglefowl^2,6^. As Japanese ornamental chickens phenotypically differ from each other, intensive artificial selection may have been conducted before these varieties diverged from ancestral Shamo in Okinawa^2,6^. We also investigated the degree of genetic differentiation among Shamo chickens by focusing on the genes encoding dopamine receptors D2, D3, and D4 in domesticated chicken populations^7^. The dopamine receptors receive the neurotransmitter when it is released from presynaptic nerve terminals. They trigger crucial physiological responses regulating movement, cognition, reward, and hormone release. Genetic differentiation was evaluated using the nucleotide differentiation (*N*_ST_) index. We found that the *N*_*ST*_ of *DRD4* (dopamine receptor D4 gene) in Shamo (0.072) was significantly higher than that of the other *DRD* genes. The genes responsible for aggressiveness, behaviour, and other traits were analysed by array comparative genomic hybridisation (aCGH) in culturally domesticated chickens, gamecocks, and ornamental chickens^1^. The assay revealed 782 gene probe candidates for artificial selection pressure in culturally domesticated chickens.

Here, the aim was to examine the effects of neurotransmitters and their receptors in the brain on Shamo strength and aggression under high-stress conditions used for artificial selection. To the best of our knowledge, no previous research has explored the stress response in Shamo from different perspectives. Moreover, there has been no study on the neurotransmitters in the brains of Shamo gamecocks. This information will increase our understanding of the mechanism underlying the aggressive behaviour of Shamo gamecocks. It may also help elucidate the roles of neurotransmitters in the human brain and under stress-related conditions such as panic disorder, depression, syncope, and anxiety^8–12^.

## Results

### Analysis of monoamines in the chicken brain

We examined four neurotransmitters and six metabolites that are believed to influence aggressive behaviour. We compared Shamo cocks selected for cockfighting with Shaver Brown cocks selected for egg laying. The neurotransmitters examined were dopamine (DA), epinephrine (Epi), norepinephrine (NE), and 5-hydroxytryptamine (5-HT) as well as their metabolites 3,4-dihydroxyphenylacetic acid (DOPAC), 5-hydroxyindoleacetic acid (5-HIAA), homovanillic acid (HVA), 3-methoxy-4-hydroxyphenylglycol (MHPG), 3-methoxytyramine (3-MT), and normethanephrine (NM). These monoamines are important substances for identifying the metabolic pathway of epinephrine^13–16^.

**Figure 1:**
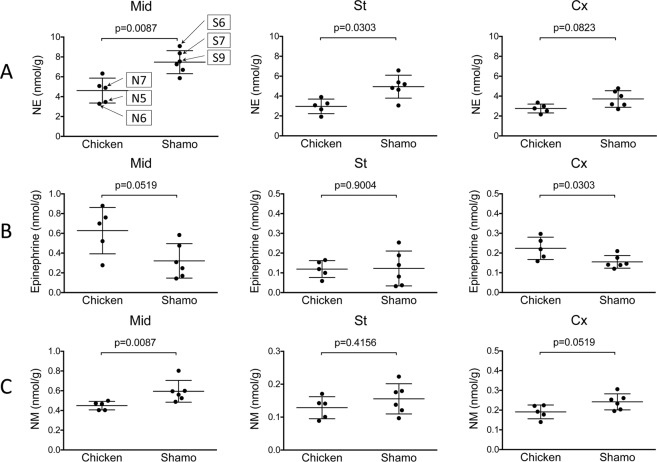
Brain neurotransmitter and metabolite concentrations in Shamo and Shaver Brown chickens. (**A**): NE; norepinephrine (St, Cx, and Mid); (**B**): epinephrine (St, Cx, and Mid); (**C**): NM; normetanephrine (St, Cx, and Mid). Error bars indicate standard deviation.

**Figure 2:**
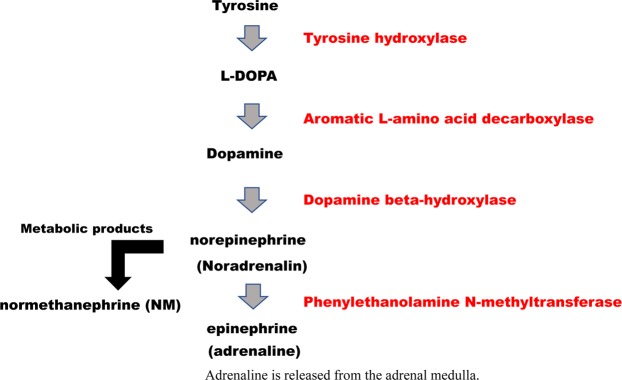
Norepinephrine biosynthesis. NE biosynthesis from dopamine proceeds via dopamine-β-hydroxylase.

The levels of NE and NM were significantly higher in Shamo than in Shaver Brown as determined using the Mann–Whitney *U* test (Figs. 1A–C and S1D–J; Tables S1–S3). The striatum (St; *P* = 0.0303) and midbrain (Mid; *P* = 0.0087) levels of NE in Shamo were significantly higher (~1.7× and ~1.6×, respectively) than those in Shaver Brown. The NE level in the central cortex (Cx) was also approximately 1.4 times higher in Shamo than in Shaver Brown; however, this difference was not significant (*P* = 0.0823) (Fig. 1A). In contrast, the Cx (*P* = 0.0303) and Mid (*P* = 0.0519) levels of Epi in Shaver Brown were significantly higher (~1.5× and ~1.9×, respectively) than those in Shamo (Fig. 1B). There were no significant differences between Shamo and Shaver Brown in terms of the levels of the other monoamines in the St, Cx, and Mid. The Mid of the Shamo brain contained a significantly higher NM level than that of the Shaver Brown brain (*P* = 0.0087) (Fig. 1C). Thus, NE biosynthesis from dopamine proceeds via dopamine-β-hydroxylase (Fig. 2), and Shamo has a higher dopamine-β-hydroxylase activity than Shaver Brown.

### Analysis of polymorphisms related to adrenergic receptor genes

There were significant differences between Shamo and Shaver Brown in terms of their brain Epi and NE levels. Therefore, we analysed the genes encoding the receptors for these neurotransmitters. We sampled three Shamo individuals with the highest NE levels and three Shaver Brown birds with the lowest NE levels (Fig. 1A). We then sequenced these samples to localise mutation sites in the *ADRα1A*, *ADRα1B*, *ADRα1D*, *ADRα2A*, *ADRα2B*, *ADRα2C*, *ADRβ1*, *ADRβ2*, and *ADRβ3* receptor genes (Tables 1 and S4).

**Table 1:** DNA and amino acid mutation sites in adrenergic receptor genes *ADRα2A*, *ADRα2B*, *ADRα2C*, *ADRα1A*, *ADRα1B*, *ADRα1D*, *ADRβ1*, *ADRβ2*, *and ADRβ3* in Shamo and Shaver Brown.

Gene name	Shaver Brown (Control chicken)						Shamo (Gamecock)					
	N5	TMHMM	N6	TMHMM	N7	TMHMM	S6	TMHMM	S7	TMHMM	S9	TMHMM
ADR*α*1A exon2	S365G	inside	S365G	Inside	S365G	inside	—		—		—	
ADR*α*1B exon1	—		R258Q	Inside	—		—		—		—	
ADR*α*1B exon2	V494M	inside	V494M	Inside	V494M	inside	V494M	inside	V494M	inside	V494M	inside
ADR*α*1D exon1	—		—		—		—		L58W	Tmhelix	—	
ADR*α*1D exon2	—		—		—		T440N	inside	T440N	inside	T440N	inside
ADR*α*2A	—		V58I	Tmhelix	V58I	Tmhelix	V58I	Tmhelix	V58I	Tmhelix	V58I	Tmhelix
	—		D273E	Inside	—		D273E	inside	D273E	inside		
	—		—		—		—		—		V296I	inside
ADR*α*2B	—		—		—		—		V292M	outside	V292M	outside
	—		—		—		R138Q	inside	—		—	
	—		—		—		R210H	inside	—		—	
ADR*β*1	—		Q403R	Inside	Q403R	inside	—		—		—	
ADR*β*2	—		—		—		T277M	Tmhelix	—		—	
	—		—		—		—		A15T	outside	A15T	outside
	—		—		—		—		T44I	Tmhelix	T44I	Tmhelix
	—		—		—		—		Q232R	inside	Q232R	inside
ADR*β*3	R342C	inside	R342C	Inside	R342C	inside	R342C	inside	R342C	inside	R342C	inside
	S396P	inside	S396P	Inside	S396P	inside	S396P	inside	S396P	inside	S396P	inside
	Q404L	inside	Q404L	Inside	Q404L	inside	Q404L	inside	Q404L	inside	Q404L	inside

A: alanine; C: cysteine; D: aspartic acid; E: glutamic acid; G: glycine; H: histidine; I: isoleucine; L: leucine; M: methionine; N: asparagine; P: proline; Q: glutamine; R: arginine; S: serine; T: threonine; V: valine; TMHMM: tool to predict transmembrane region.

The results confirmed the presence of 54 *ADR* mutations in the three Shamo and three Shaver Brown birds, among which 33 were identified in Shamo and 21 in Shaver Brown. Many of the mutations were localised to *ADRα2A*, *ADRα2B*, and *ADRβ2*. The Shamo-specific mutations included the following: V296I in *ADRα2A* (bird S9), R138Q and R210H (bird S6), V292M in *ADRα2B* (birds S7 and S9), and L58W in *ADRα1D* (bird S7) (Table 1). In addition, the S6, S7, and S9 Shamo birds carried the original T440N mutation site in ADRα1D (exon 2). For *ADRβ2*, Shamo-specific mutations A15T (G43R), T44I (C131Y and T132Y), Q232R (A695R), and T277M (C830Y) were detected (Table 1). Therefore, Shamo-specific mutations were found in *ADRα1D* (T440N), *ADRα2B* (V292M), and *ADRβ2* (A15T, T44I, and Q232R).

The N5, N6, and N7 Shaver Brown birds carried the original mutation S365G in *ADRα1A* (exon 2). Bird N6 had the R258Q site in *ADRα1B* and D273E site in *ADRα2A*. Bird N7 had the original R68Q mutation site in *ADRα2B*. Shaver Brown-specific mutations were detected in *ADRα1A* (S365G), *ADRα1B* (Q258R), and *ADRβ1* (Q403R).

Shamo and Shaver Brown had the same original mutations, namely, V494M in *ADRα1B* and V58I and D273E in *ADRα2a*. R342C, S396P, and Q404L of *ADRβ3* were observed in both breeds.

### Prediction of transmembrane helices in proteins encoded by the *ADR* genes

**Figure 3:**
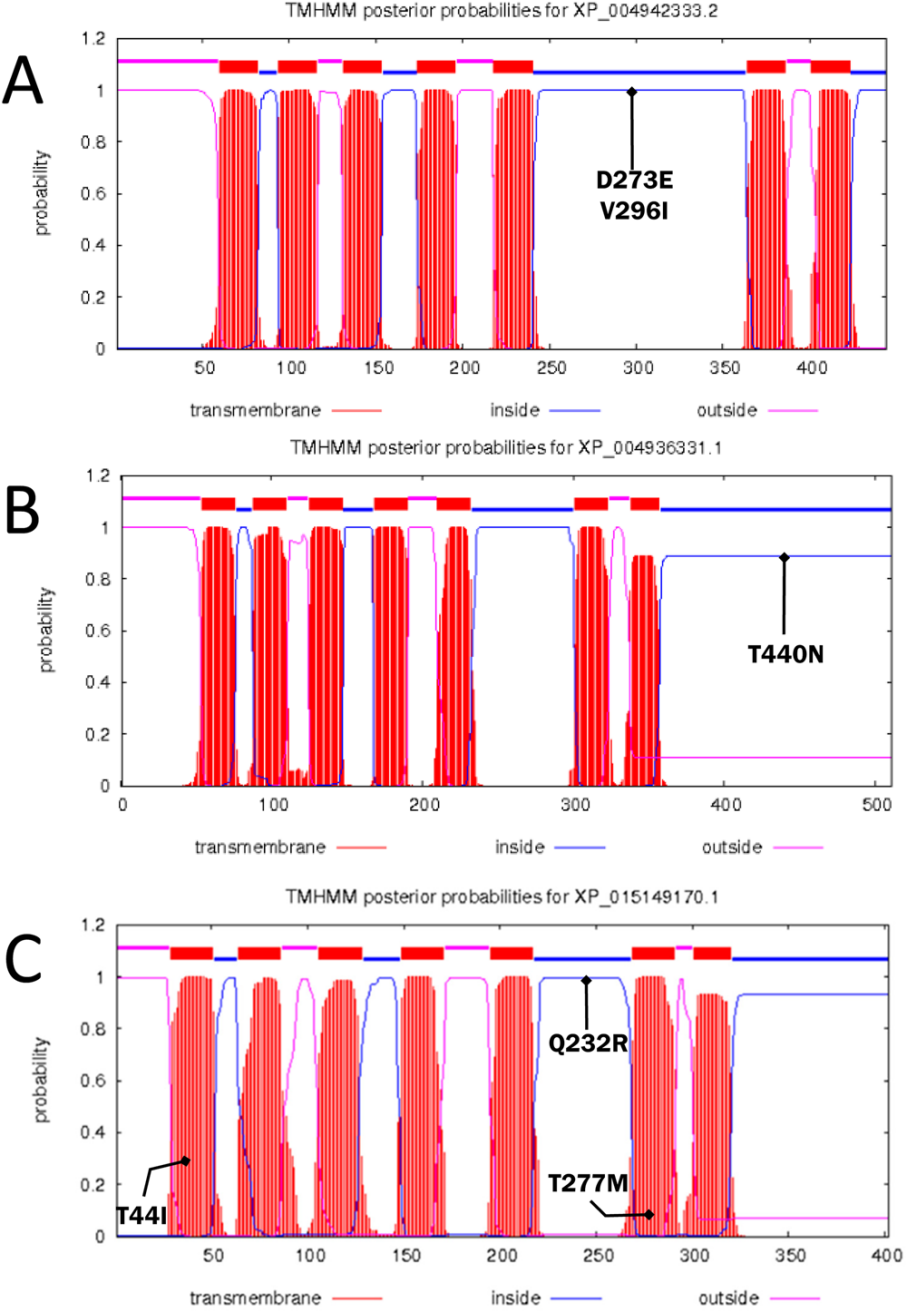
Prediction of transmembrane helices in proteins encoded by *ADRα2A*, *ADRα1D*, and *ADRβ2*. Transmembrane helices in the proteins encoded by *ADRα2A* (**A**), *ADRα1D* (**B**), and *ADRβ2* (**C**) in Shamo were predicted using the TMHMM secondary structure. Segments of the cytoplasmic side (intracellular), exterior (extracellular), and transmembrane region (TMhelix) are displayed.

We analysed transmembrane helices in the proteins encoded by each mutant allele using TMHMM server. The *ADR* genes encode membrane proteins. Each of these proteins has seven transmembrane structures (Fig. 3A–C; Table S5A-C). The TMHMM analysis indicated that the most highly mutated gene was ADRβ2: T277M, T44I (Fig. 3C; Table S5C). The mutated sectors were located in the TMhelix (transmembrane region), whereas Q232R was intracellular on the cytoplasmic side. A15T was extracellular and not directly related to behaviour.

Several mutations were observed in D273E and V296I of *ADRα2A* (Fig. 3A; Table S5A). In *ADRα2B*, mutations were detected at the R138Q and R210H sites in bird S6 and the V292M site in birds S7 and S9. The mutations at the R138Q and R210H sites were intracellular on the cytoplasmic side and possibly related to behaviour. As the V292M site was extracellular, it was probably not associated with bird behaviour. The T440N site of *ADRα1D* was also found in Shamo (Fig. 3B; Table S5B). The differences in the number of mutations in this membrane protein suggest that *ADRβ2* is more closely associated with fighting behaviour than ADRα2. T 441 I (TMhelix) and Q232R (inside) of *ADRβ2* may regulate fighting behaviour (Fig. 3C; Table S5C). The TMHMM analysis disclosed that Shaver Brown birds N5, N6, and N7 had mutations on intracellular *ADRα1A* (S365G) and *ADRβ1* (Q403R). These mutations conferred the birds a selective advantage as egg layers. Furthermore, *ADRα1B* (V494M), *ADRα2A* (V58I and D273E), and *ADRβ3* (R342C, S396P, and Q404L) mutated simultaneously with breeder domestication.

### Molecular phylogeny analysis of aggressiveness in Shamo based on *ADRα1D, ADRα2A*, and *ADRβ2*

**Figure 4:**
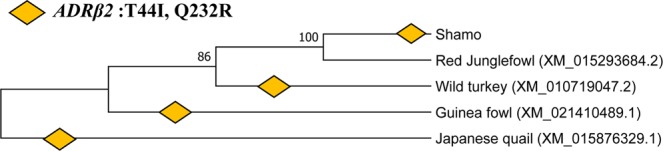
Phylogenetic tree of *ADRβ2* in Galliformes members. *ADRβ2*: T44I and Q232R were confirmed in wild turkey, Guinea fowl, and Japanese quail (Fig. 4). Red junglefowl was the only species lacking these three mutations. Diamonds indicate the T44I and Q232R mutations.

We conducted an evolutionary analysis to identify the origin of aggressiveness. We constructed phylogenetic trees using *ADRα1D(1536* *bp)*: T440N, *ADRα2A(1332* *bp)*: D273E, V296I, and *ADRβ2(1170* *bp)*: T44I, Q232R. We also extracted sequences of red junglefowl, wild turkey, Guinea fowl, and Japanese quail from the NCBI database^17,18^. These species exhibit strong aggressive behaviours. For example, pheasants can be territorial^19–21^. The evolutionary analysis indicated that these birds have similar mutations. The *ADRα1D*: T440N, *ADRα2A*: D273E and V296I mutations appeared exclusively in Shamo and not in any other Phasianidae species. In contrast, *ADRβ2*: T44I and Q232R were confirmed in wild turkey, Guinea fowl, Japanese quail, and Shamo (Fig. 4). Hence, red junglefowl was the only species that lacked these two mutations.

## Discussion

Here, we examined the effects of brain neurotransmitters on Shamo gamecock behaviour. In this breed, artificial selection under extreme stress has improved bird strength and aggression. We analysed 10 brain neurotransmitters and the *ADR* genes that may either regulate aggression in Shamo used for cockfights or determine calmness of Shaver Brown bred for egg production under high population densities.

We observed significant differences between Shamo and Shaver Brown in terms of Mid NE level. The Mid NE levels were found to be closely related to Shamo aggressiveness. The Mid NE level in S6 was four times higher than that in N5 (Fig. 1A). NE is secreted from the ends of the sympathetic nerves in response to fighting stimulation. It serves as an alarm system in the brain and attenuates stress and tension caused by sudden unpleasant internal and external stimuli. The NE alarm system reduces anxiety and fear^22–26^. NE increases attention, concentration, judgment, and motivation. It is also known as the ‘angry hormone’ as it induces corporeal tension or excitement in response to stress^23,27,28^. Aggressiveness increases as negative emotions intensify. NE also increases the heart rate and blood flow to the brain and skeletal muscles^29^. Therefore, excess NE increases nerve activity, and thus, the animal becomes restless and aggressive^30^. A portion of the Mid is related to the pathway of sensory and motor neurons^31–34^, maintains reflex eyeball movements, and adjusts iris contraction^35^.

We analysed the receptor genes (*ADR*) regulated by Epi or NE (Table 1)^36–39^. In both breeds, 54 amino acid mutation sites were confirmed in eight *ADR* genes (Shamo, 33 sites; Shaver Brown, 21 sites). Prediction of transmembrane helices in proteins (TMHMM) suggested an association between fighting behaviour and specific mutations, namely, T440N of *ADRα1D*; V296I and D273E of *ADRα2A*; and T44 I, Q232R, and T277M of *ADRβ2* (Fig. 3A–C). Several mutations may be related to domestication including *ADRα1B* (V494M), *ADRα2A* (V58I), and *ADRβ3* (R342C, S396P, and Q404L) in domesticated chickens originally selected from the wild type (*G. gallus*). The mutation sites in *ADRα1A* (S365G) may be important for selecting traits associated with calmness and non-aggressiveness. Thus, these mutations may not be related to fighting behaviour, but are advantageous for housed birds raised for human food production. Moreover, T44I and Q232R of ADR*β2* have been detected in other Galliformes members using the molecular phylogeny analysis. We could not confirm these mutations in the wild type (*G. gallus*) used in our previous study^1,7^.

The wild type still maintains a substantial proportion of ancestral polymorphisms at the genomic level^40^. Our phylogenetic analysis revealed that Shamo are distinctively aggressive and that this trait evolved by combining mutations derived from artificial selection and natural adaptation in Galliformes (Fig. 4).

The vascular smooth muscle relaxes in response to the ADRβ subunit (Gs) and contracts in response to the ADRα subunit (Gi). This mechanism substantially affects the blood vessel responses during sympathetic excitement. In this state, the ADRα subunit (Gi) is expressed mainly in the blood vessels that regulate blood flow to the heart and to the skeletal muscles required for fighting^41–44^. The blood vessels of the heart, lungs, and skeletal muscles mainly express the ADRβ subunit (Gs)^12^ and expand in response to sympathetic excitation^45–54^. In this way, they maintain blood flow to the organs required for fighting. Therefore, relative differences in NE level and specific receptor mutations were observed in different environments, and they favoured aggression in Shamo and a calm nature in Shaver Brown living communally. These mutations occurred in captive populations and were deemed necessary for domestication^55,56^.

Changes in the NE receptor genes and hormone sensitivity occur as the NE level increases^57^. Excess NE increases the aggressiveness of Shamo, whereas low NE levels result in lethargy but enable Shamo to endure prolonged fighting stress.

Although dopamine is the precursor of NE, we observe no significant differences in the brains of the different breeds in terms of the dopamine levels. However, in our previous study, we found that the *N*_*ST*_ of *DRD4* in Shamo (0.072) was significantly higher than that of the other genes in domesticated chicken populations^7^. Therefore, the fighting behaviour of Shamo is more closely related to the norepinephrine level than the dopamine level, and mutations in the *ADR* receptor have the strongest influence on fighting behaviour. Thus, the accumulation of several *ADR* polymorphisms and elevation in the brain NE levels may have conserved the aggressiveness in Shamo.

NE also reduces pain perception^58–62^. *ADRα2A*, closely associated with pain response, was mutated in Shamo bred for cockfights^63,64^. The Mid NE alleviates pain via the *ADRα2* receptor gene^58,63,64^. In combats, Shamo birds either fight or fly away (‘fight-or-flight’). They must be able to take immediate action in response to changes in the NE level^55,65^. If Shamo birds were insensitive to pain during fighting, they could either continue fighting or escape. Several receptors may be involved in stress relief. Isolation of mutant alleles in Shamo by aCGH revealed the γ-aminobutyric acid (GABA-a) receptor gene associated with the central nervous system (CNS)^1^. The GABA-a receptor mitigates anxiety and relaxes skeletal muscles^66–68^. Artificial selection of Shamo for cockfight enhanced its fighting behaviour by reducing stress related to anxiety and fear.

The CNS responds to stimuli from receptors and induces feelings or sensations. This response may be used to assess the effects of neurotransmitters on the brain. The ‘fight-or-flight’ response is a conserved behaviour in vertebrates experiencing fear, stress, or intense physical activity. Thus, we can investigate the effects of the neurotransmitters triggering the ‘fight-or-flight’ response in the brain^69^.

On the basis of the typical brain monoamine levels, we propose that aggressiveness may be related to the *ADR* genes based on the observed relative differences in brain NE level. Aggressiveness is determined by several genes and neurotransmitters^70–76^. Identification of these factors may help elucidate their modes of action.

In future research, we will investigate the gene levels of receptors for catecholamines and other substances in various parts of the brains of Shamo and other chickens. We will also measure neurotransmitter levels after cockfights and analyse the exomes of ornamental chickens, Shamo, and other chickens bred for different purposes. We will explore the relationships between chicken domestication and neurotransmitters and the effect of artificial selection on the genes implicated in this process. Here, we identified the adrenergic genes in Shaver Brown crucial for group living and those related to the combative trait in Shamo. Therefore, we will conduct *ex vivo* assays of the localised NE levels in the Mid of live animals and/or animal models subjected to various stressors.

Stress contributes to numerous human diseases and influences brain neurotransmitter levels. In the future, we will examine the effects of neurotransmitters that govern the ‘fight-or-flight’ response. Elucidation of the neurochemical mechanisms involved in this process may help improve defences against the deleterious effects of stress.

## Conclusions

Our results showed that the Mid level of NE was significantly higher in Shamo bred for fighting than in Shaver Brown bred for egg laying. The fighting instinct might be correlated with the type and distribution of *ADRβ2* alleles and their responses to the NE level. Gene polymorphisms can change with the NE level. Therefore, we believe that the strong fighting behaviour of Shamo was probably due to the combination of naturally inherited genes with mutant genes derived from artificial selection.

## Materials and Methods

### Ethics statements

All animal experiments were conducted in strict compliance with the ethical guidelines of Tokai University, Japan. Approval was also obtained from the Animal Investigation Committee of Tokai University, Japan (Approval Nos. 141024 and 152010).

### Chickens and brain samples

**Figure 5:**
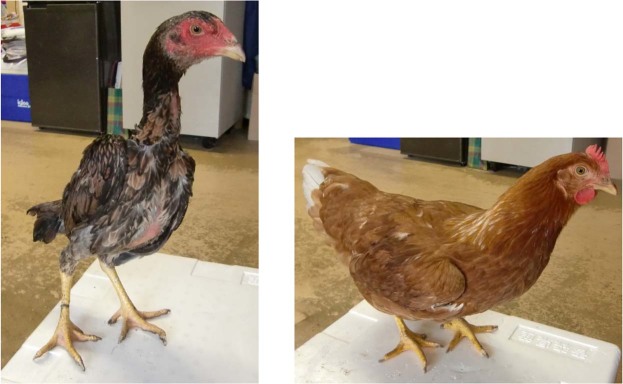
Characteristics of female Shamo and Shaver Brown chickens. Left: Shamo. Right: Shaver Brown.

**Figure 6:**
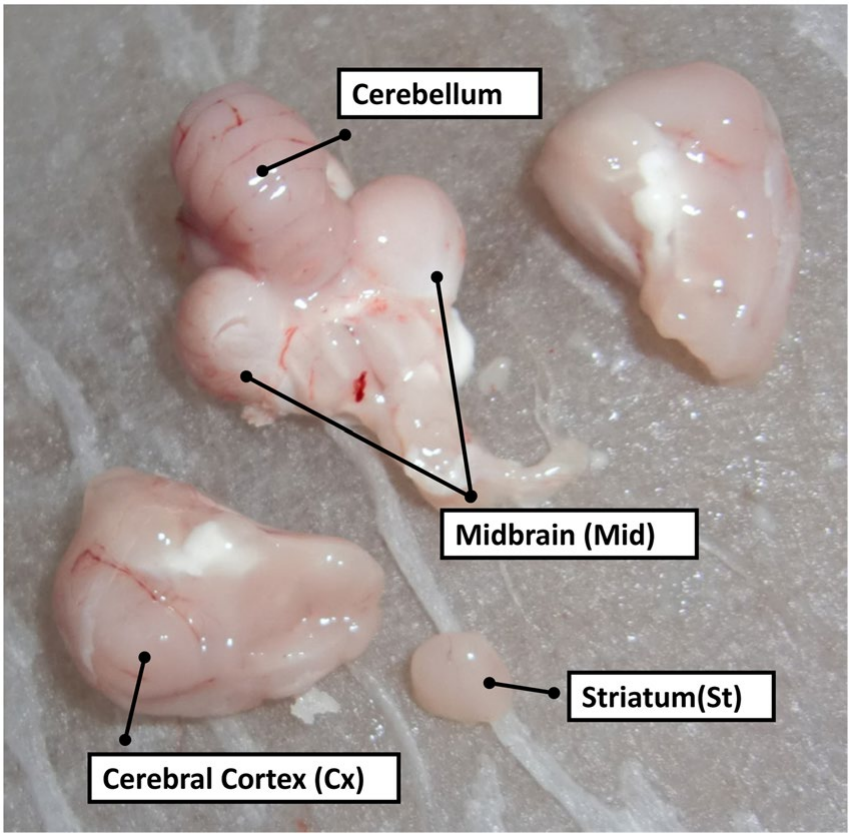
Striatum (St), cerebral cortex (Cx), and midbrain (Mid) of Shamo. Shamo skulls were more difficult to remove than Shaver Brown skulls as the former have been strengthened for cockfighting by selective breeding. Thus, the removal of their skulls required considerable force and resulted in the skulls shattering into numerous fragments. Moreover, the Shamo brains had a higher tension and gloss than the Shaver Brown chicken brains.

Shamo chicks were randomly selected from various areas and breeders. These birds would have otherwise been used in cockfights. The Shaver Brown chicks were randomly selected from several farms. Shaver Brown was selected as the docile breed. It has a superior egg production trait. Furthermore, it was previously described as a strong candidate for artificial selection. The brain samples were randomly collected from 6 female Shamo birds and 5 domesticated Shaver Brown chickens. The latter were bred as egg layers and served as the controls^77^ (Fig. 5). Shamo females were used because they are invaluable in the genetic improvement of chicken lines^2^. Shamo birds were individually maintained in separate cages. Shaver Brown chickens were maintained in groups outside. Samples were obtained from 24-wk-old chickens, because at this age, the females start ovulating and the males are trained for cockfights. The chickens were decapitated and the backs of their skull were opened with pliers. Then, the brains were rapidly excised and divided into Cx, St, and Mid according to the Chicken Brain Atlas (http://avianbrain.org/nomen/Chicken_Atlas.html)^78–81^. These tissues were selected because they are easily distinguished from each other and respond to multiple neurotransmitters. The brain samples were frozen on dry ice, weighed, and stored at −80 °C. Images of Shamo brains are shown in Fig. 6.

The skull of Shamo birds was more difficult to remove than that of Shaver Brown chickens as the former birds have been strengthened for cockfighting by selective breeding. Thus, the removal of their skull required considerable force and resulted in their skulls shattering into numerous fragments. Moreover, the brain of Shamo birds had a higher tension and gloss than the brain of Shaver Brown birds (Fig. 6).

### Analysis of monoamines and their metabolites in the brain tissues

The brain tissues were homogenised in 0.2 M perchloric acid (HClO_4_) containing 100 µM EDTA-Na and 100 ng isoproterenol as an internal standard. The homogenates were centrifuged at 20 000 × *g* and 4 °C for 15 min. The pH of the supernatant was adjusted to 3.0 with 1 M sodium acetate. The samples were passed through a 0.45-µm filter (UFC40HV; EMD Millipore, Billerica, MA, USA). The filtrate (10 µL) was injected into a high-performance liquid chromatography-electrochemical detection (HPLC-ECD) system (Eicom, Kyoto, Japan) consisting of a 150 mm × 3 mm octadecylsilane column (EICOMPAK SC-5ODS; Eicom, Kyoto, Japan), a pump (EP-300; Eicom, Kyoto, Japan), a column oven (ATC-300; Eicom, Kyoto, Japan), and an electrochemical detector (ECD-300; Eicom, Kyoto, Japan). The mobile phase consisted of aceto–citric acid buffer (0.1 M; pH 3.5), methanol, sodium-1-octane sulfonate (0.46 M), and disodium ethylenediaminetetraacetic acid (0.015 mM) [830:170:1.9:1]. The flow rate was 0.5 mL min^−l^. The levels of DA, Epi, NE, 5-HT, DOPAC, 5-HIAA, HVA, MHPG, 3-MT, and NM were calculated using PowerChrom v. 2.6.11 (eDAQ Inc., Colorado Springs, CO, USA).

### DNA extraction, polymerase chain reaction (PCR) amplification, and sequencing of the adrenaline genes (*ADRα1A*, *ADRα1B*, *ADRα1D*, *ADRα2A*, *ADRα2B*, *ADRα2C*, *ADRβ1*, *ADRβ2*, and *ADRβ3*)

Blood was drawn from each chicken and the samples were suspended in 400 μL of TNES-8M urea. Twenty microlitres of proteinase K (20 mg mL^−l^) and 20 μL of 1 M dithiothreitol (DTT) were added to the samples, which were then incubated for 1–5 h at 60 °C and mixed with 500 μL of phenol/chloroform/isoamyl alcohol solution (25:24:1) for 3 min. This step was repeated twice. After precipitation with 2–2.5 volumes of ethanol, the pellets were rinsed in 70% v/v cold ethanol and dried. The samples were dissolved in TE buffer [10 mM Tris-HCl (pH 8.0) and 1 mM EDTA]. The PCR was performed to amplify *ADRα1A* (Ex1 and Ex2), *ADRα1B* (Ex1 and Ex2), *ADRα1D* (Ex1 and Ex2), *ADRα2A*, *ADRα2B*, *ADRα2C*, *ADRβ1*, *ADRβ2*, and *ADRβ3* (Ex1 and Ex2). The PCR primers are listed in Table S6. The PCR enzymes used were KOD-Plus and KOD FX neo (TOYOBO, Osaka, Japan). The PCR was performed under the following conditions: denaturation for 20 s at 98 °C, 30 cycles for 5 s at 98 °C, annealing at 64 °C for 30 s, 68 °C for 1 min, and a final extension at 68 °C for 7 min. The PCR products were purified using the MinElute PCR purification kit (QIAGEN, Duesseldorf, Germany) and ExoSAP-IT (USB Corp., Cleveland, OH, USA) and sequenced using BigDye Terminator v. 3.1 (Thermo Fisher Scientific, Waltham, MA, USA) and the ABI Prism 3730xl DNA sequencer (ABI, Foster City, CA, USA).

### Sequence assembly and alignment

The contigs of each *ADR* gene were assembled from the ABI DNA sequences using ATGC GENETYX v. 13 (GENETYX Corp., Tokyo, Japan). The sequences were then determined and registered in the International Nucleotide Sequence Database Collaboration (INSDC). The accession numbers of the *ADR* DNA sequences (LC483765-LC483818) were obtained from the DDBJ/EMBL/GenBank. All assembled sequences were aligned using CLUSTALW-MEGA v. 7^82,83^. These analyses confirmed the mutation sites in the *ADR* genes of each chicken breed. The ADR receptor gene sequences were mapped for both breeds and their mutation sites were determined. The sequence of red junglefowl, the wild ancestor of chickens, was used as the reference (Table S4).

### Statistical analysis

Data are presented as mean ± SD. Statistical analyses were conducted using Prism v. 6.0c (GraphPad Software, San Diego, CA, USA). Comparisons of the mean monoamine and metabolite levels were made using the Mann–Whitney *U* test. The results with a *P* value of <0.05 were considered statistically significant.

### Molecular phylogeny analysis of complete *ADRβ2*

A phylogenetic tree (Fig. 1) was constructed using the UPGMA method^83^. The UPGMA algorithms were incorporated into CLUSTALW-MEGA v. 7 using distances corrected for multiple hits based on Kimura’s two-parameter model^83^. Sites representing gaps in any of the aligned sequences were excluded from the analysis. For phylogenetic tree construction, we used the bootstrap analysis of 1000 replications to assess statistical confidence in the branching order of the trees. Complete *ADRβ2* sequences for red junglefowl (XM_015293684.2), wild turkey (XM_010719047.2), Guinea fowl (XM_021410489.1), Japanese quail (XM_015876329.1), and Shamo (LC483807 - LC483809) were obtained from the DDBJ/EMBL/GenBank database. These birds were selected because of their close evolutionary relationships with chickens.

### Prediction of transmembrane helices in proteins

The transmembrane helices in the encoded proteins were analysed for each mutant allele using TMHMM server v. 2.0 (http://www.cbs.dtu.dk/services/TMHMM/).

For this analysis, *ADRα2A*; XP_004942333.2, *ADRα1D*; XP_004936331.1, and *ADRβ2*; XP_015149170.1 were used as the reference from the NCBI.

## Supplementary information

Supplementary Information.

## Data Availability

All sequence data are available from the DDBJ, EBI, and NCBI databases.
