# Supplementary material for: Analysis of the source of aggressiveness in gamecocks
Source: Sci Rep. 2020 Apr 24;10:7005. doi: 10.1038/s41598-020-63961-1 (PMC7181795; doi:10.1038/s41598-020-63961-1)
Supplement: Supplementary file 1 — Supplementary Information. [file 41598_2020_63961_MOESM1_ESM.pdf]

Scientific Reports

BIOLOGICAL SCIENCE

Analysis of the source of aggressiveness in gamecocks

Tomoyoshi Komiyama1*, Masanobu Yoshikawa1, Keiko Yokoyama2,
and Hiroyuki Kobayashi1

1 Department of Clinical Pharmacology, Tokai University School of Medicine, 143 Shimokasuya, Isehara, Kanagawa 259-1193, Japan
2 Support Center for Medical Research and Education, Tokai University, 143 Shimokasuya, Isehara, Kanagawa 259-1193, Japan

***Corresponding author**
E-mail: komiyama@tokai-u.jp

Supplementary Information

Table Fig. S1 (D-J), Table S1, S2, S3, S4, S5, and S6

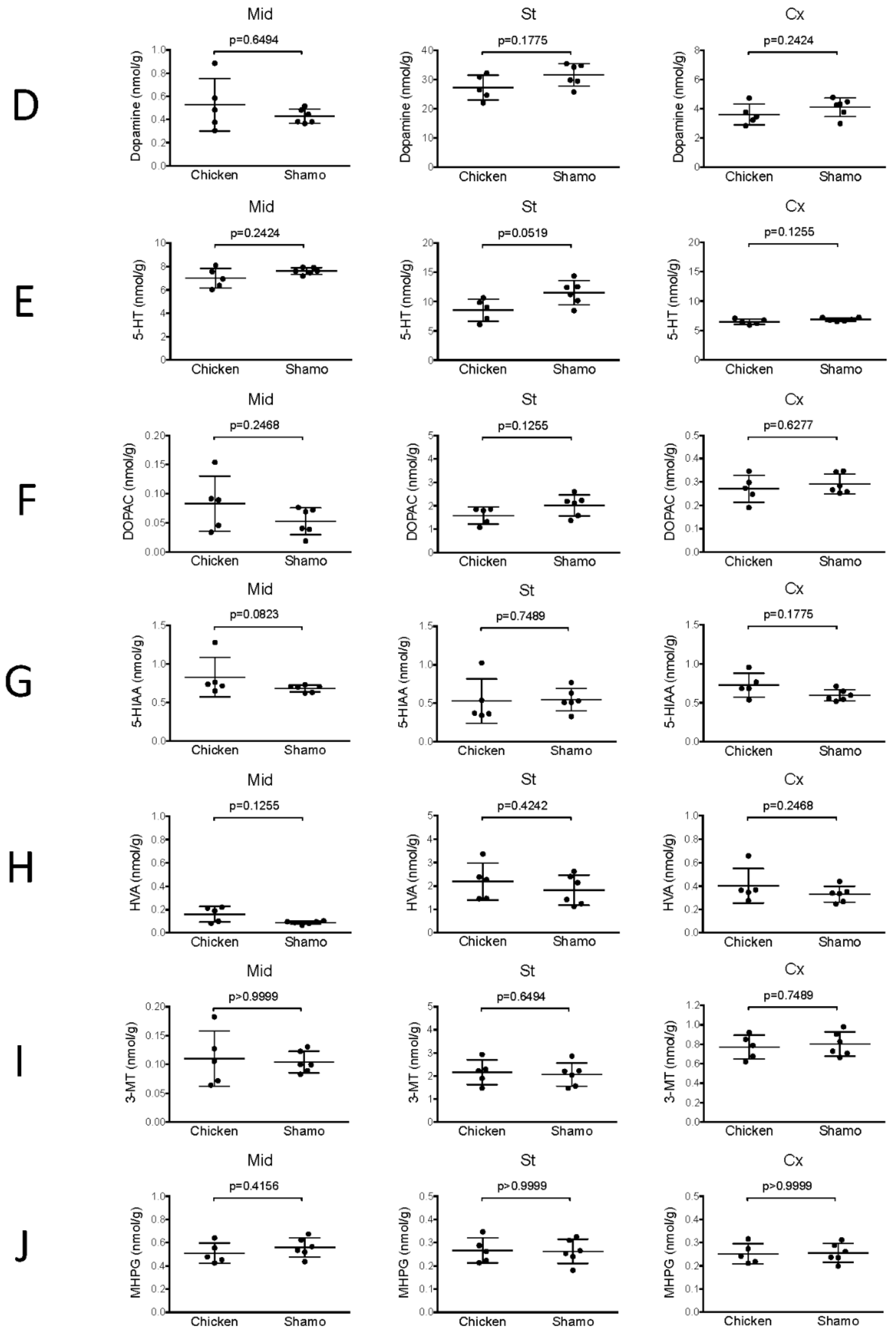

Fig. S1; D-J. Brain neurotransmitter and metabolites concentrations in Shamo and Shaver Brown (Chicken).

D: dopamine (St, Cx, and Mid); E: serotonin (5-HT) (St, Cx, and Mid) ; F: 3,4-dihydroxyphenylacetic acid (DOPAC) (St, Cx, and Mid); G: 5-hydroxyindoleacetic acid (5-HIAA) (St, Cx, and Mid); H: homovanillic acid (HVA); I: 3-methoxytyramine (3-MT) (St, Cx, and Mid); J: 3-methoxy-4-hydroxyphenylglycol (MHPG) (St, Cx, and Mid).
Error bars indicate standard deviation.

nmol/ g FW	368.38	169.18	183.2	168.15	183.2	153.18	191.19	211.26	182.15	167.24	176.22	
	MHPG	NE	Epi	DOPAC	NM	DA	5-HIAA	ISO	HVA	3MT	5HT	
Shamo#4	0.325637711	4.814220679	0.081140092	2.100144473	0.223050141	34.78901481	0.529999275	4.920482058	1.425454678	2.856825328	12.43779334	
Shamo#5	0.181006538	3.049313127	0.032521432	1.375690241	0.175771574	25.78905957	0.326909767	3.55635142	1.132561175	2.040868219	8.480196149	
Shamo#6	0.265385915	5.379084744	0.188422575	2.227623812	0.120954349	29.47520032	0.509700635	5.294746912	2.625672354	2.203895111	11.24922147	
Shamo#7	0.253700783	6.576404207	0.13991602	2.18681662	0.096892483	34.31090925	0.769314744	4.43627342	2.144231108	2.223373152	14.38763444	
Shamo#8	0.240423047	4.638667119	0.037209531	1.582030471	0.179454975	29.89766055	0.630431725	4.844937297	1.246343642	1.472335371	12.54898298	
Shamo#9	0.310238651	5.188337937	0.253703086	2.598600034	0.13770208	35.45138378	0.509196438	5.241975348	2.401790597	1.562664302	10.184717	
Chicken#4	0.287719604	3.256565864	0.153698884	1.849685582	0.170692882	26.51229083	0.371074914	4.663550482	2.272365249	2.927800288	9.013922923	
Chicken#5	0.223033748	1.922799062	0.058717362	1.073994562	0.100639293	22.02257999	0.364116244	2.889806923	1.461548181	1.47211541	6.092499538	
Chicken#6	0.213585805	2.665763349	0.164763628	1.317875133	0.088766884	24.68885083	0.341516999	4.163151926	1.471672158	1.896641779	7.091359675	
Chicken#7	0.34708732	3.07014899	0.119161666	1.797007975	0.141201432	32.25948167	1.024257155	6.370799111	3.366917287	2.29214195	9.87860366	
Chicken#8	0.262410192	3.898096921	0.099996756	1.859550281	0.142519452	30.94871546	0.537512136	4.395082395	2.387708356	2.225714017	10.65336052	
ng/ g FW												Weight (mg)
	MHPG	NE	Epi	DOPAC	NM	DA	5-HIAA	ISO	HVA	3MT	5HT	
Shamo#4	119.95842	814.4698545	14.86486486	353.1392931	40.86278586	5328.981289	101.3305613	1039.50104	259.6465696	477.7754678	2191.787942	96.2
Shamo#5	66.67918858	515.8827949	5.957926371	231.322314	32.20135237	3950.368144	62.50187829	751.3148009	206.296018	341.3148009	1494.380165	133.1
Shamo#6	97.76286353	910.033557	34.51901566	374.5749441	22.15883669	4515.011186	97.44966443	1118.568233	478.2662192	368.5794183	1982.337808	89.4
Shamo#7	93.45829428	1112.596064	25.63261481	367.7132146	17.75070291	5255.74508	147.0852858	937.2071228	390.5716963	371.836926	2535.388941	106.7
Shamo#8	88.56704197	784.7697032	6.81678608	266.0184237	32.87615148	4579.723644	120.5322416	1023.541453	227.0214944	246.2333675	2211.381781	97.7
Shamo#9	114.2857143	877.7630122	46.47840532	436.9545958	25.22702104	5430.442968	97.35326689	1107.419712	437.4861573	261.3399779	1794.750831	90.3
Chicken#4	105.9901478	550.9458128	28.15763547	311.0246305	31.27093596	4061.152709	70.94581281	985.2216749	413.91133	489.6453202	1588.433498	101.5
Chicken#5	82.16117216	325.2991453	10.75702076	180.5921856	18.43711844	3373.418803	69.61538462	610.5006105	266.2210012	246.1965812	1073.620269	163.8
Chicken#6	78.68073879	450.9938434	30.18469657	221.6007036	16.26209323	3781.838171	65.294635	879.5074758	268.0650836	317.1943712	1249.639402	113.7
Chicken#7	127.8600269	519.4078062	21.83041723	302.166891	25.86810229	4941.507402	195.8277254	1345.89502	613.2839838	383.3378197	1740.807537	74.3
Chicken#8	96.66666667	659.4800371	18.31940576	312.6833798	26.1095636	4740.724234	102.7669452	928.5051068	434.9210771	372.2284123	1877.33519	107.7
	Raw Data											
	MHPG	NE	Epi	DOPAC	NM	DA	5-HIAA	ISO	HVA	3MT	5HT	
Shamo#4	11.54	78.352	1.43	33.972	3.931	512.648	9.748	100	24.978	45.962	210.85	
Shamo#5	8.875	68.664	0.793	30.789	4.286	525.794	8.319	100	27.458	45.429	198.902	
Shamo#6	8.74	81.357	3.086	33.487	1.981	403.642	8.712	100	42.757	32.951	177.221	
Shamo#7	9.972	118.714	2.735	39.235	1.894	560.788	15.694	100	41.674	39.675	270.526	
Shamo#8	8.653	76.672	0.666	25.99	3.212	447.439	11.776	100	22.18	24.057	216.052	
Shamo#9	10.32	79.262	4.197	39.457	2.278	490.369	8.791	100	39.505	23.599	162.066	
Chicken#4	10.758	55.921	2.858	31.569	3.174	412.207	7.201	100	42.012	49.699	161.226	
Chicken#5	13.458	53.284	1.762	29.581	3.02	552.566	11.403	100	43.607	40.327	175.859	
Chicken#6	8.946	51.278	3.432	25.196	1.849	429.995	7.424	100	30.479	36.065	142.084	
Chicken#7	9.5	38.592	1.622	22.451	1.922	367.154	14.55	100	45.567	28.482	129.342	
Chicken#8	10.411	71.026	1.973	33.676	2.812	510.576	11.068	100	46.841	40.089	202.189	

Table S1. The raw data of neurotransmitters in the striatum (St) from six Shamos and five Shaver Browns.
Neurotransmitter concentrations in striata (St) of six Shamo and five Shaver Brown (Chicken; 4-8).

nmol/ g FW	368.38	169.18	183.2	168.15	183.2	153.18	191.19	211.26	182.15	167.24	176.22	
	MHPG	NE	Epi	DOPAC	NM	DA	5-HIAA	ISO	HVA	3MT	5HT	
Shamo#4	0.236822113	2.692371941	0.139257114	0.257355368	0.305976444	2.996663839	0.598744828	0.555119472	0.341651027	0.979372836	6.752130537	
Shamo#5	0.198906655	3.183129261	0.145910173	0.346582656	0.253206217	4.328527757	0.711786379	0.627535959	0.336560641	0.904913083	7.253656947	
Shamo#6	0.261813475	4.783808467	0.120530814	0.342588002	0.260758934	4.788064819	0.648619155	0.624308064	0.440545253	0.826189825	6.848566519	
Shamo#7	0.288748007	4.012613005	0.140963855	0.252139909	0.203534323	3.779931254	0.519026771	0.753502665	0.269063242	0.728344543	6.635661098	
Shamo#8	0.234488953	3.139985745	0.209769807	0.26617206	0.195805379	4.492590946	0.544786804	0.730817313	0.247943366	0.707469039	6.510925304	
Shamo#9	0.311964244	4.466130433	0.17595656	0.283875172	0.233173005	4.277825863	0.556181802	0.702091922	0.352003692	0.664442292	7.197086272	
Chicken#4	0.241401188	2.770365347	0.158803609	0.272479587	0.224654794	3.772436022	0.683838744	0.663699347	0.365031378	0.922208981	6.430572336	
Chicken#5	0.21774992	2.512133439	0.182231697	0.246552944	0.177924307	3.473204319	0.76608322	0.647361015	0.367622988	0.676594833	6.204898666	
Chicken#6	0.211041357	2.168835832	0.297124805	0.190415395	0.13983104	2.857406604	0.683039967	0.700429674	0.276424351	0.620407969	6.750882001	
Chicken#7	0.316119179	2.983173418	0.261829127	0.345561159	0.192109664	4.748350819	0.954859681	0.603686231	0.658755684	0.849939364	7.08404821	
Chicken#8	0.273559563	3.345832878	0.219486155	0.297751377	0.221483697	3.240303468	0.538585664	0.620868801	0.34743715	0.786345339	5.928725278	
ng/ g FW												Weight (mg)
	MHPG	NE	Epi	DOPAC	NM	DA	5-HIAA	ISO	HVA	3MT	5HT	
Shamo#4	87.24053008	455.4954849	25.51190337	43.27430515	56.05488448	459.0289668	114.4740237	117.2745397	62.23173449	163.7903131	1189.860443	852.7
Shamo#5	73.27323346	538.5218083	26.73074374	58.27787353	46.38737903	663.0438817	136.0864378	132.5732467	61.30452075	151.3376641	1278.239427	754.3
Shamo#6	96.4468478	809.3247164	22.08124505	57.60617251	47.77103667	733.4357689	124.0094962	131.8913216	80.24531786	138.1719863	1206.854392	758.2
Shamo#7	106.3689908	678.8538682	25.82457816	42.39732569	37.28748806	579.0098695	99.23272843	159.1849729	49.00986947	121.8083413	1169.336199	628.2
Shamo#8	86.38104061	531.2227883	38.42982862	44.75683187	35.87154547	688.1750811	104.1577891	154.3924656	45.16288405	118.3171221	1147.355257	647.7
Shamo#9	114.9213883	755.5799466	32.23524177	47.7336102	42.71729457	655.2773658	106.3363987	148.3239395	64.11747256	111.121329	1268.270543	674.2
Chicken#4	88.9273696	468.6904094	29.09282109	45.81744251	41.15675827	577.8617499	130.7431296	140.2131239	66.49046551	154.2302299	1133.195457	713.2
Chicken#5	80.21471554	425.0027352	33.38484683	41.45787746	32.59573304	532.0254376	146.4674508	136.761488	66.96252735	113.1537199	1093.427243	731.2
Chicken#6	77.74341521	366.923646	54.43326428	32.01834862	25.61704646	437.6975437	130.5904114	147.972773	50.35069547	103.7570287	1189.640426	675.8
Chicken#7	116.4519832	504.6932789	47.96709603	58.10610891	35.1944905	727.3523785	182.5596225	127.5347532	119.9923479	142.1438592	1248.350976	784.1
Chicken#8	100.773872	566.0480063	40.20986359	50.06689402	40.57581322	496.3496852	102.9721931	131.1647429	63.28567681	131.5083945	1044.759969	762.4
	Raw Data											
	MHPG	NE	Epi	DOPAC	NM	DA	5-HIAA	ISO	HVA	3MT	5HT	
Shamo#4	74.39	388.401	21.754	36.9	47.798	391.414	97.612	100	53.065	139.664	1014.594	
Shamo#5	55.27	406.207	20.163	43.959	34.99	500.134	102.65	100	46.242	114.154	964.176	
Shamo#6	73.126	613.63	16.742	43.677	36.22	556.091	94.024	100	60.842	104.762	915.037	
Shamo#7	66.821	426.456	16.223	26.634	23.424	363.734	62.338	100	30.788	76.52	734.577	
Shamo#8	55.949	344.073	24.891	28.989	23.234	445.731	67.463	100	29.252	76.634	743.142	
Shamo#9	77.48	509.412	21.733	32.182	28.8	441.788	71.692	100	43.228	74.918	855.068	
Chicken#4	63.423	334.27	20.749	32.677	29.353	412.131	93.246	100	47.421	109.997	808.195	
Chicken#5	58.653	310.762	24.411	30.314	23.834	389.017	107.097	100	48.963	82.738	799.514	
Chicken#6	52.539	247.967	36.786	21.638	17.312	295.796	88.253	100	34.027	70.119	803.959	
Chicken#7	91.31	395.73	37.611	45.561	27.596	570.317	143.145	100	94.086	111.455	978.832	
Chicken#8	76.83	431.555	30.656	38.171	30.935	378.417	78.506	100	48.249	100.262	796.525	

Table S2. The raw data of neurotransmitters in the cerebral cortex (Cx) from six Shamos and five Shaver Browns. Neurotransmitter concentrations in cerebral cortices (Cx) of Shamo and Shaver Brown (Chicken; 4-8).

nmol/ g FW	368.38	169.18	183.2	168.15	183.2	153.18	191.19	211.26	182.15	167.24	176.22	
	MHPG	NE	Epi	DOPAC	NM	DA	5-HIAA	ISO	HVA	3MT	5HT	
Shamo#4	0.51921344	6.703041492	0.169803157	0.01884994	0.803221615	0.365038641	0.630067435	2.703314529	0.080139297	0.130345444	7.93132721	
Shamo#5	0.436689503	5.863833692	0.247207987	0.06883638	0.598873722	0.380905285	0.702076128	2.053580798	0.095937712	0.122623728	7.913816071	
Shamo#6	0.535062987	9.088998227	0.30834695	0.040441204	0.595111928	0.44676484	0.70588204	2.230680367	0.105401422	0.10003289	7.462664215	
Shamo#7	0.623109291	8.36455869	0.582982945	0.076186305	0.52549822	0.481825862	0.620823284	2.441208736	0.086667515	0.088843425	7.191535687	
Shamo#8	0.566271716	7.257452849	0.142857143	0.038891957	0.56002634	0.381582411	0.7304958	3.0053992	0.063579206	0.082990702	7.58662062	
Shamo#9	0.67416805	7.53870432	0.475613069	0.071822561	0.488101185	0.517030519	0.706365809	2.495257638	0.097355273	0.099384002	7.615737911	
Chicken#4	0.476812034	5.075305749	0.699671871	0.091269851	0.467337049	0.584515726	0.763056699	1.676169879	0.190963461	0.127189945	6.946273056	
Chicken#5	0.452685745	3.465201258	0.520714591	0.045299644	0.404066533	0.375936148	0.714564472	2.112228353	0.101543821	0.071721151	6.031287915	
Chicken#6	0.42529899	3.286293441	0.276700558	0.033759244	0.403957288	0.303710589	0.648985836	2.354976985	0.080055391	0.063959085	6.390954093	
Chicken#7	0.64107836	4.891715555	0.761124506	0.088912667	0.499275008	0.483556692	1.276903991	2.030675135	0.221530499	0.105326223	8.102489376	
Chicken#8	0.554908217	6.327406747	0.879093689	0.15349989	0.472116117	0.88587785	0.736971184	1.713795706	0.21421261	0.18243542	7.555252958	
ng/ g FW												Weight (mg)
	MHPG	NE	Epi	DOPAC	NM	DA	5-HIAA	ISO	HVA	3MT	5HT	
Shamo#4	191.2678469	1134.02056	31.10793832	3.169617362	147.1501999	55.91661907	120.4625928	571.1022273	14.59737293	21.79897202	1397.658481	175.1
Shamo#5	160.867679	992.0433839	45.28850325	11.57483731	109.7136659	58.34707158	134.2299349	433.8394794	17.47505423	20.50759219	1394.572668	230.5
Shamo#6	197.1065033	1537.67672	56.48916117	6.800188501	109.0245052	68.43543827	134.9575872	471.2535344	19.19886899	16.72950047	1315.070688	212.2
Shamo#7	229.5410005	1415.116039	106.8024755	12.81072718	96.27127385	73.80608561	118.6952037	515.7297576	15.78648788	14.85817432	1267.292419	193.9
Shamo#8	208.6031746	1227.815873	26.17142857	6.53968254	102.5968254	58.45079365	139.6634921	634.9206349	11.58095238	13.87936508	1336.914286	157.5
Shamo#9	248.3500264	1275.397997	87.13231418	12.07696363	89.42013706	79.19873484	135.0500791	527.1481286	17.73326305	16.6209805	1342.045335	189.7
Chicken#4	175.648017	858.6402266	128.1798867	15.3470255	85.61614731	89.53611898	145.8888102	354.1076487	34.78399433	21.27124646	1224.072238	282.4
Chicken#5	166.7603748	586.2427488	95.39491299	7.617135207	74.02498884	57.58589915	136.6175814	446.2293619	18.49620705	11.99464525	1062.833556	224.1
Chicken#6	156.6716418	555.9751244	50.69154229	5.676616915	74.00497512	46.52238806	124.079602	497.5124378	14.58208955	10.69651741	1126.21393	201
Chicken#7	236.1604462	827.5804376	139.4380094	14.95066495	91.46718147	74.07121407	244.1312741	429.000429	40.35178035	17.61475761	1427.820678	233.1
Chicken#8	204.4170891	1070.470673	161.0499638	25.81100652	86.4916727	135.698769	140.9015206	362.0564808	39.01882694	30.51049964	1331.386676	276.2
	Raw Data											
	MHPG	NE	Epi	DOPAC	NM	DA	5-HIAA	ISO	HVA	3MT	5HT	
Shamo#4	33.491	198.567	5.447	0.555	25.766	9.791	21.093	100	2.556	3.817	244.73	
Shamo#5	37.08	228.666	10.439	2.668	25.289	13.449	30.94	100	4.028	4.727	321.449	
Shamo#6	41.826	326.295	11.987	1.443	23.135	14.522	28.638	100	4.074	3.55	279.058	
Shamo#7	44.508	274.391	20.709	2.484	18.667	14.311	23.015	100	3.061	2.881	245.728	
Shamo#8	32.855	193.381	4.122	1.03	16.159	9.206	21.997	100	1.824	2.186	210.564	
Shamo#9	47.112	241.943	16.529	2.291	16.963	15.024	25.619	100	3.364	3.153	254.586	
Chicken#4	49.603	242.48	36.198	4.334	24.178	25.285	41.199	100	9.823	6.007	345.678	
Chicken#5	37.371	131.377	21.378	1.707	16.589	12.905	30.616	100	4.145	2.688	238.181	
Chicken#6	31.491	111.751	10.189	1.141	14.875	9.351	24.94	100	2.931	2.15	226.369	
Chicken#7	55.049	192.909	32.503	3.485	21.321	17.266	56.907	100	9.406	4.106	332.825	
Chicken#8	56.46	295.664	44.482	7.129	23.889	37.48	38.917	100	10.777	8.427	367.729	

Table S3. The raw data of neurotransmitters in the midbrain (Mid) from five Shamos and six Shaver Browns. Neurotransmitter concentrations in midbrains of Shamo (4-9) and Shaver Brown (Chicken; 4-8).

	GeneName	Shaver Brown			Shamo			
		N5	N6	N7	S6	S7	S9	Reference Sequence
1	ADR α1A exon1	none	none	none	none	none	none	XP_015152983.1, XM_015297497.1, ENSGALE00000309612
2	ADR α1A exon2	S365G(A1093R)	S365G (A1093R)	S365G (A1093R)	none	none	none	XM_015297497.1, ENSGALE00000003480
3	ADR α1B exon1	none	R258Q (G773R)	none	none	none	none	XM_414483.4, XM_004944816.2, ENSGALE00000012976
4	ADR α1B exon2	V494M (G1480A)	V494M (G1480A)	V494M (G1480R)	V494M (G1480A)	V494M (G1480A)	V494M (G1480A)	XM_414483.4, ENSGALE00000012977
5	ADR α1D exon1	none	none	none	none	L58W (T173K)	none	XM_004936274.2, XM_420871.3
6	ADR α1D exon2	none	none	none	none	none	none	XM_004936274.2, XM_420871.3
7	ADR α1D exon2	none	none	none	T440N (C1319M)	T440N (C1319M)	T440N (C1319M)	XM_004936274.2, XM_420871.3
8	ADR α2A	none	V58I (G172R), D273E (C819M)	V58I (G172R)	V58I (G172A), D273E (C819M)	V58I (G172A), D273E (C819M)	V58I (G172R), V296I (G886R)	XM_004942276.2, ENSGALG00000023521
9	ADR α2B	none	none	none	R138Q (G413R), R210H (G629R)	V292M (G874R)	V292M (G874R)	XM_025154909.1, XM_021410469.1, XM_010718981.2,
10	ADR α2C	none	none	none	none	none	none	XM_004936302.3, XM_015876372.1
11	ADR β1	none	Q403R (A1208G)	Q403R (A1208G)	none	none	none	XM_015288961.1, ENSGALE00000096864
12	ADR β2	none	none	none	T277M (C830Y)	A15T (G43R),T44I (C131Y, T132Y), Q232R (A695R)	A15T (G43R), T44I (C131Y, T132Y), Q232R (A695R)	XM_015293684.2, ENSGALE00000364971
13	ADR β3	R342C (C1024Y)	R342C (C1024Y)	R342C (C1024Y)	R342C (C1024Y)	R342C (C1024Y)	R342C (C1024Y)	XM_428541.6
14	ADR β3	S396P (T1186Y)	S396P (T1186Y)	S396P (T1186Y)	S396P (T1186Y)	S396P (T1186Y)	S396P (T1186Y)	XM_428541.6
15	ADR β3	Q404L (A1211W)	Q404L (A1211W)	Q404L (A1211W)	Q404L (A1211W)	Q404L (A1211W)	Q404L (A1211W)	XM_428541.6

Hetero sites are included in these numbers.

A: alanine; C: cysteine; D: aspartic acid; E: glutamic acid; G: glycine; H: histidine; I: isoleucine; L: leucine; M: methionine; N: asparagine; P: proline; Q: glutamine; R: arginine; S: serine; T: threonine; V: valine

Table S4. DNA and amino acid mutation sites on the *ADR* by breed.
54 amino acid mutation sites were confirmed for eight *ADR* genes (Shamo, 33 sites; Shaver Brown, 21 sites). Hetero sites are included in these numbers.

Table S5 (A) Transmembrane helices in proteins encoded by each mutant allele extracted alpha-2A adrenergic receptor.			
# XP_004942333.2 Length: 444			
# XP_004942333.2 Number of predicted TMHs: 7			
# XP_004942333.2 Exp number of AAs in TMHs: 157.45425			
# XP_004942333.2 Exp number, first 60 AAs: 1.74334			
# XP_004942333.2 Total prob of N-in: 0.00113			
XP_004942333.2	TMHMM2.0	outside	1 59
XP_004942333.2	TMHMM2.0	TMhelix	60 82
XP_004942333.2	TMHMM2.0	inside	83 93
XP_004942333.2	TMHMM2.0	TMhelix	94 116
XP_004942333.2	TMHMM2.0	outside	117 130
XP_004942333.2	TMHMM2.0	TMhelix	131 153
XP_004942333.2	TMHMM2.0	inside	154 173
XP_004942333.2	TMHMM2.0	TMhelix	174 196
XP_004942333.2	TMHMM2.0	outside	197 217
XP_004942333.2	TMHMM2.0	TMhelix	218 240
XP_004942333.2	TMHMM2.0	inside	241 363
XP_004942333.2	TMHMM2.0	TMhelix	364 386
XP_004942333.2	TMHMM2.0	outside	387 400
XP_004942333.2	TMHMM2.0	TMhelix	401 423
XP_004942333.2	TMHMM2.0	inside	424 444

Table S5 (B) Transmembrane helices in proteins encoded by each mutant allele extracted alpha-1D adrenergic receptor.			
# XP_004936331.1 Length: 511			
# XP_004936331.1 Number of predicted TMHs: 7			
# XP_004936331.1 Exp number of AAs in TMHs: 154.20159			
# XP_004936331.1 Exp number, first 60 AAs: 7.55847			
# XP_004936331.1 Total prob of N-in: 0.00003			
XP_004936331.1	TMHMM2.0	outside	1 53
XP_004936331.1	TMHMM2.0	TMhelix	54 76
XP_004936331.1	TMHMM2.0	inside	77 87
XP_004936331.1	TMHMM2.0	TMhelix	88 110
XP_004936331.1	TMHMM2.0	outside	111 124
XP_004936331.1	TMHMM2.0	TMhelix	125 147
XP_004936331.1	TMHMM2.0	inside	148 167
XP_004936331.1	TMHMM2.0	TMhelix	168 190
XP_004936331.1	TMHMM2.0	outside	191 209
XP_004936331.1	TMHMM2.0	TMhelix	210 232
XP_004936331.1	TMHMM2.0	inside	233 300
XP_004936331.1	TMHMM2.0	TMhelix	301 323
XP_004936331.1	TMHMM2.0	outside	324 337
XP_004936331.1	TMHMM2.0	TMhelix	338 357
XP_004936331.1	TMHMM2.0	inside	358 511

Table S5 (C) Transmembrane helices in proteins encoded by each mutant allele extracted beta-2 adrenergic receptor.			
# XP_015149170.1 Length: 402			
# XP_015149170.1 Number of predicted TMHs: 7			
# XP_015149170.1 Exp number of AAs in TMHs: 153.60987			
# XP_015149170.1 Exp number, first 60 AAs: 23.00243			
# XP_015149170.1 Total prob of N-in: 0.00236			
# XP_015149170.1 POSSIBLE N-term signal sequence			
XP_015149170.1	TMHMM2.0	outside	1 28
XP_015149170.1	TMHMM2.0	TMhelix	29 51
XP_015149170.1	TMHMM2.0	inside	52 63
XP_015149170.1	TMHMM2.0	TMhelix	64 86
XP_015149170.1	TMHMM2.0	outside	87 105
XP_015149170.1	TMHMM2.0	TMhelix	106 128
XP_015149170.1	TMHMM2.0	inside	129 148
XP_015149170.1	TMHMM2.0	TMhelix	149 171
XP_015149170.1	TMHMM2.0	outside	172 194
XP_015149170.1	TMHMM2.0	TMhelix	195 217
XP_015149170.1	TMHMM2.0	inside	218 268
XP_015149170.1	TMHMM2.0	TMhelix	269 291
XP_015149170.1	TMHMM2.0	outside	292 300
XP_015149170.1	TMHMM2.0	TMhelix	301 320
XP_015149170.1	TMHMM2.0	inside	321 402

Table S5. Transmembrane helices in proteins encoded by each mutant allele.
A: alpha-2A adrenergic receptor. NCBI reference sequence: XP_004942333.2.
B: alpha-1D adrenergic receptor. NCBI reference sequence: XP_004936331.1.
C: beta-2 adrenergic receptor. NCBI reference sequence: XP_015149170.1.

Gene Name	Primer No.	Forward (5'−3')	Primer No.	Reverse (5'-3')
ADRα1A Ex1	F437	TGGTGGCTATTAGCTGTGCTAGA	R1767	TTCCCTTTGAACCAGACCTGCTTG
ADRα1A Ex2	F3	AGGCTTCGAGTCATAAATTTCCCA	R758	TATTTGTTTCATCCCAACCCCTTCA
ADRα1B Ex1	F35	AACTGAACTCAACCATCTGCGTGTT	R1209	TTCACTTCCCACCCTTTCATCTGGT
ADRα1B Ex2	F57	AATTGAAAGCAGCACATGTTAGGGA	R2188	TGGCTTCCTTCTAGTGGCATACTG
ADRα1D Ex1	F9	GCTGGGAGATCACTCCATCCCTACT	R1291	TCACTGCTGGTTTCTTGACCGATCT
ADRα1D Ex2	F20	ACACTAAGGAAGCTCCCACACCT	R389	GGTAGCCTTTGGAAGGGCTGAATCC
ADRα1D Ex2	F40	TGGCTGTCCACCAGCCTTGCTACAC	R2307	AATCTGCCTGACAGACCCACAGA
ADRα2A	F102	TAGGAGCGCGGAGGTCCGCGGAGA	R1636	TCACAAGCTGAAGGAAACCATCCCAT
ADRα2B_1	F238	AGTGCCCCAATACCACGGTGGAAGCT	R904	GCAGGATGAGGACGGCCACCAGGAT
ADRα2B_2	F880	ATCCTGGTGGCCGTCCTCATCCTGC	R1793	CGCTTCTGGAAGTACCTTGACTTCA
ADRα2C	F9	TCCGCGGGGCTCTGTAGGACGGCG	R1463	GCACAGAGCCTTTCCCTTCCATTTTAA
ADR β1	F888	AACGCCGTGCGCTGCACACCGAGGAGG	R2618	TGGGCTTCCTGTTTGCCTTAGGTTC
ADRβ2	F1	ACCTGCTGTCCCCTATGCGCGGA	R2	GCCATTGGGGTTTAGGTGTGCT
ADRβ3 Ex1_1	F757	TGCAGCCTCGACGGCGGCGGCCGC	R1859	AGCACAGCAGCTTGCGGAAAGCGCT
ADRβ3 Ex1_2	F1478	TCCACCATCTCCTTCTACGTGCC	R2216	GCCCGCTCACCAGCTCTGCATTCA
ADRβ3 Ex2	F2443	TACTGGGCCACCAGGAGTGTGTTC	R2704	CTGCTGCAGGGACAGGGGTGGCCC

Table S6. Primers used for amplifying ADR genes.
The primer pairs were designed to cover the complete region for ADR genes.
PCR was performed under the following conditions: denaturation for 20 s at 98 °C, 30 cycles of 5 s at 98 °C, annealing at 64 °C for 30 s, 68 °C for 1 min, and a final extension at 68 °C for 7 min.
